# Ischemic heart disease and primary care: identifying gender-related differences. An observational study

**DOI:** 10.1186/1471-2296-9-60

**Published:** 2008-10-30

**Authors:** Inés Cruz, Catalina Serna, Jordi Real, Gisela Galindo, Eduardo Gascó, Leonardo Galván

**Affiliations:** 1 Primary Care Research Institute IDIAP Jordi Gol, Catalan Institute of Health, Lleida, Spain; 2 Ronda Health Center, Catalan Institute of Health, Lleida, Spain; 3 University of Lleida, Lleida, Spain; 4 Regional Primary Care Management Office, Catalan Institute of Health, Lleida, Spain; 5 Catalan Health Department, Lleida, Spain

## Abstract

**Background:**

Gender-related differences are seen in multiple aspects of both health and illness. Ischemic heart disease (IHD) is a pathology in which diagnostic, treatment and prognostic differences are seen between sexes, especially in the acute phase and in the hospital setting. The objective of the present study is to analyze whether there are differences between men and women when examining associated cardiovascular risk factors and secondary pharmacological prevention in the primary care setting.

**Methods:**

Retrospective descriptive observational study from January to December of 2006, including 1907 patients diagnosed with ischemic heart disease in the city of Lleida, Spain. The clinical data were obtained from computerized medical records and pharmaceutical records of medications dispensed in pharmacies with official prescriptions. Data was analyzed using bivariate descriptive statistical analysis as well as logistic regression.

**Results:**

There were no gender-related differences in screening percentages for arterial hypertension, diabetes, obesity, dyslipemia, and smoking. A greater percentage of women were hypertensive, obese and diabetic compared to men. However, men showed a tendency to achieve control targets more easily than women, with no statistically significant differences. In both sexes cardiovascular risk factors control was inadequate, between 10 and 50%. For secondary pharmaceutical prevention, the percentages of prescriptions were greater in men for anticoagulants, beta-blockers, lipid-lowering agents and angiotensin-converting enzyme inhibitors/angiotensin II receptor blockers, with age group variations up to 10%. When adjusting by age and specific diagnoses, differences were maintained for anticoagulants and lipid-lowering agents.

**Conclusion:**

Screening of cardiovascular risk factors was similar in men and women with IHD. Although a greater percentage of women were hypertensive, diabetic or obese, their management of risk factors tended to be worse than men. Overall, a poor control of cardiovascular risk factors was noted.

Taken as a whole, more men were prescribed secondary prevention drugs, with differences varying by age group and IHD diagnosis.

## Background

Ischemic heart disease (IHD) is considered to be responsible for approximately half of deaths in the Western Hemisphere, in both men and women, even though global prevalence of this disease is lower in women. In Spain the incidence of IHD is among the lowest in the world. Projects such as REGICOR (Girona Coronary Register) [1] or WHO-MONICA-Catalunya [2] analyzed the standardized annual incidence of acute myocardial infarction (AMI), obtaining figures of 31–39 new cases per 100,000 women and 178–210 cases per 100,000 men.

The majority of patients with this pathology are over 65. Above this age, prevalence increases rapidly among women until it becomes the primary cause of death. In fact, the incidence of infarct in women between 60–70 years old is the same as that of men ten years younger, between 50–60 years old [3].

For a long time women have been invisible to the health care system, to diagnosis processes and even to treatment. This situation is known as Yentl syndrome. Women's health problems have been reduced to social, cultural, psychological and reproductive causes that have hidden their physiology, their condition and their environment.

IHD is one of the diseases that most clearly shows biological and gender inequalities: in diagnosis, treatment, prevention and rehabilitation. Previous studies show that there are important differences between men and women in the clinical management of IHD, especially in patients admitted with acute coronary pathologies: women arrive an hour later to the hospital on the average, have more co morbidity, progress to more severe conditions and have a greater risk of adjusted mortality at 28 days [4]. With regard to diagnostic tests, other research has shown that women wait longer to be visited and to get an electrocardiogram, and are referred less often for coronary angiographies. Furthermore, revascularization and pharmacological treatments at discharge are different, with men being prescribed beta blockers and anticoagulants more frequently [3].

Recently, a study done in the United Kingdom in a large population diagnosed with angina showed that there are also differences in primary care follow-up, in screening and management of cardiovascular risk factors (CVRF), and in the prescription of medication recommended for secondary prevention [5].

In this context, the present study was proposed with the following objective: to evaluate gender-related differences in clinical follow-up of ischemic heart disease in a primary care setting, both for detection and management of the principal CVRF and the use of recommended medications for secondary prevention.

## Methods

This was a retrospective descriptive observational study using data from a clinical registry. The study period was from January to December of 2006. During this period, the study scope (the city of Lleida, Spain) had a population of 144,521 inhabitants, assigned to any of its basic health areas (BHA). Those BHA belong to the Catalan Institute of Health, the public institution which provides primary and specialized health care services and prescription drug coverage to 97% of the city population. All practices have been computerized since 2003 and share the same information system, which made it possible to create a comprehensive database from primary care records. Analytic results, pharmaceutical prescription information from specialists and hospital discharge diagnoses were also available. All patients registered with a diagnosis of ischemic heart disease (codes I20 – I25 of the ICD-10) in the computerized primary care medical records by the 31^st ^of December 2006 were included in the study: this represents 1907 individuals, of whom 1266 were men and 641 women.

Being an observational retrospective study, no ethical approval was considered necessary.

### Variables

The data obtained from the clinical records were anonymized. Variables studied were: sex, age, diagnosis of hypertension, diagnosis of diabetes, diagnosis of obesity, diagnosis of dyslipemia, diagnosis of smoking, arterial pressure, basal blood glycemia, HbA1c, body mass index (BMI), total and fractional cholesterol, optimal management of systolic and diastolic blood pressure (SBP < 140 and DBP < 90), optimal glycosylated hemoglobin management (HbA1c < 7 mg), optimal management of BMI (BMI < 25), optimal cholesterol management (LDL < 100) and prescribing of medications recommended for secondary prevention: beta-blockers, antiplatelet/anticoagulants, lipid-lowering agents and angiotensin converting enzyme inhibitors (ACE inhibitors)/angiotensin II receptor blockers (ARB). Variables reflect the most recent available data recorded during 2006. A CVRF was considered screened if the variable value was filled in during the study period. Annual screening findings and previously registered CVRF were gathered to produce CVRF year-prevalence.

### Data sources

Demographic and clinical data were obtained from the primary care information system and the Catalan Health Institute's computerized primary care medical records system (SIAP/e-CAP), which registers administrative, clinical and treatment data on all users assigned to the BHAs. Information relating to pharmaceutical prescriptions was obtained from the pharmacy unit at the Catalan Health Service, from databases of all prescribed medications purchased by patients in pharmacies, irrespective of prescriber.

### Statistical Analysis

Once the two databases were linked, a descriptive study of all the included qualitative variables was produced as frequency tables and percentages. Quantitative variables were described using means, medians and standard deviations. The chi-squared test was used to determine statistically significant differences between sexes in the distribution of the various factors studied. In addition, age and IHD diagnoses adjusted odds ratios (OR), with their respective 95% confidence intervals (95% CI), were estimated using generalized additive models (GAM) with logit link [6]. P-values less than 0.01 were considered significant.

## Results

### Characteristics of patients diagnosed with IHD in Lleida: sex, age and diagnoses

A total of 144,521 persons made up the population assigned to the BHAs in the city of Lleida, of whom 1907 were registered in the SIAP/e-CAP system with a diagnosis of IHD. This is equivalent to a global prevalence of 1.32% (0.88% in women and 1.76% in men). Mean age of the study population was 71.5 years (SD = 12). The mean age for men was 69.2 (SD = 12), while mean age for women was 76 (SD = 11).

**Figure 1:**
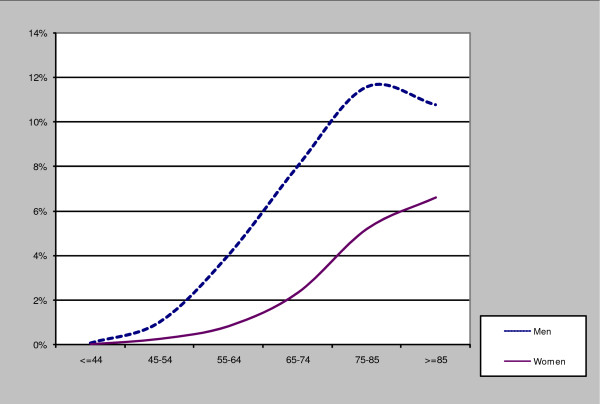
Prevalence of IHD by age and sex.

Table 1 and Figure 1 show the prevalence of IHD grouped by age and sex.

**Table 1:** IHD prevalence rates by age and sex

	**MEN** *(N = 1266)*		**WOMEN** *(N = 641)*		**TOTAL** *(N = 1907)*
**Age Groups**	**N**	**%**	**N**	**%**	**%**

≤ 44	37	0.08	5	0.01	0.05

45–54	97	1.02	23	0.25	0.64

55–64	262	4.03	57	0.83	2.38

65–74	355	8.00	131	2.32	4.82

75–84	405	11.57	273	5.19	7.74

> 84	110	10.77	152	6.60	7.88

**Age (mean ± SD)**	69.2 ± 12.0		76.0 ± 10.7		71.5 ± 12.0

The most frequent diagnoses registered by sex were as follows: for men, chronic ischemic heart disease (55%), acute myocardial infarction (25%), angina (16%) and other ischemic diseases (4%). For women: chronic ischemic heart disease (56%), angina (23%), acute myocardial infarction (17%) and other ischemic diseases (4%).

### Prevalence, screening and management of CVRF by gender

An evaluation was done as to whether patients with IHD were screened for the principal modifiable CVRF and percentages by sex are presented in Table 2. Screening percentages for blood pressure, smoking and BMI were similar between men and women, although they were slightly higher in men. On the other hand, cholesterol and basal glycemia screening percentages were slightly higher in women. No observed differences were statistically significant.

**Table 2:** Percentage of patients screened for the following risk factors: HTN, dyslipemia, smoking, obesity, and diabetes

	**WOMEN** *(n = 641)*	**MEN** *(n = 1266)*	**TOTAL** *(n = 1907)*
**Blood pressure**	83.5	85.4	84.7

**Cholesterol**	71.8	68.8	69.8

**Smoking**	85.7	88.3	87.4

**BMI**	70.2	73.6	72.5

**Glycemia**	64.3	60.5	61.8

Annual screening findings and previously registered CVRF produced CVRF prevalence by sex (Table 3). Women had more hypertension (HTN), obesity and diabetes mellitus (DM), while men were more likely to be smokers. Rates of dyslipemia were similar in both groups.

**Table 3:** Detected prevalence of modifiable cardiovascular risk factors

	**WOMEN** *(n = 641)*	**MEN** *(n = 1266)*	**TOTAL** *(n = 1907)*	
	**%**	**%**	**N**	**%**

Hypertension *	70.8	57.1	1177	61.7

**Dyslipemia**	44.0	44.2	842	44.2

Smoker *	3.1	9	261	13.7

Obesity *	27.3	21.9	452	23.7

**Diabetes Mellitus**	36.8	32.4	646	33.9

* Distributions of risk factors with a p-value < 0.01 in the chi-squared test between men and women.

The level of management of CVRF with respect to the current established recommendations can be seen in Table 4, differentiated by sex.

**Table 4:** Management of risk factors associated with ischemic heart disease as suggested in clinical practice guidelines, in percentage

**TARGET**	**WOMEN**	**MEN**	**TOTAL**
**SBP < 140 and DBP < 90**, n = 1177	49.8	53.5	52.1

**LDL < 100**, n = 842	36.2	39.8	38.6

**BMI < 25**, n = 1907	9.7	11.1	10.6

**HbA1c < 7**, n = 646	33.1	35.1	34.4

The Chi-square test between men and women did not detect any statistical differences

Management was assessed for the total number of patients with each risk factor, except for BMI, which was calculated for all the patients with IHD. When data about CVRF control was not registered, the patient was considered not controlled for that item.

The percentage of men that achieved recommended management objectives is greater than that of women, for all CVRF. These differences are not statistically significant.

Risk factor control was low for both sexes: 52% of hypertensive patients with IHD had their blood pressure under 140/90 and this percentage dropped to 10.6% when considering patients whose body mass index was below 25.

### Use of recommended medications for secondary prevention

Table 5 shows the percentage of patients who were dispensed at least one prescription, for each of the four pharmacological groups that have shown effectiveness in secondary prevention, by sex. Dispensation percentages were greater in men and all differences were statistically significant except those for angiotensin-converting enzyme inhibitors/angiotensin II receptor blockers.

**Table 5:** Percentage of consumption of medication containers by sex

**Containers (> = 1)**	**WOMEN** *(n = 641)*	**MEN** *(n = 1266)*	**TOTAL** *(n = 1907)*
Anticoagulants*	72.9	83.8	80.1

Beta-blockers*	43.8	51	48.6

**ACE inhibitors/ARB**	45.1	49.8	48.2

Lipid-lowering agents*	55.7	68	63.9

*p-value < 0.01 in the chi-squared test between men and women.

Table 6 shows the unadjusted odds ratio for men with respect to women. Differences were statistically significant in all pharmacological groups, except for ACEinhibitors/ARB.

**Table 6:** Non-adjusted and age/diagnosis adjusted odds ratio for pharmacological secondary prevention in men with respect to women

	**OR_unadj_**	**C.I. 95%**	**OR_adj_**	**C.I. 95%**
**Any medication**	1.63*	(1.24–2.13)	1.43*	(1.07–1.92)

**Anticoagulants**	1.93*	(1.53–2.43)	1.81*	(1.41–2.31)

**Beta blockers**	1.33*	(1.10–1.62)	1.03	(0.84–1.27)

**ACE inhibitors/ARB**	1.20	(0.99–1.45)	1.15	(0.94–1.42)

**Lipid-lowering agents**	1.70*	(1.39–2.05)	1.24*	(1.01–1.53)

*p-value < 0,05 in the model adjusted for age and IHD specific diagnosis (chronic ischemic heart disease, acute myocardial infarction and angina)

Estimated age-adjusted OR using GAM link logit

As previous results showed that age and diagnosis had a different distribution in men and women and that prescribing is related to both variables, odds ratios were recalculated adjusted for age and specific IHD diagnosis to control the potential confounding effect of these variables. Statistically significant differences persisted for antiplatelet/anticoagulant drugs and lipid-lowering agents.

**Figure 2:**
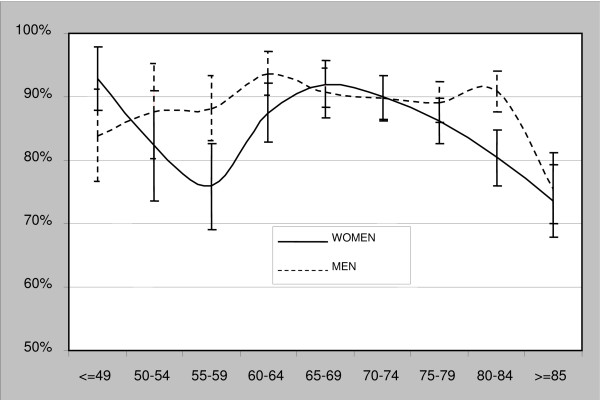
Percentage (95% CI) of patients with any prescribed medication, by age and sex.

All differences vary by age group. In the 55–59 and 80–84 groups there are differences in pharmaceutical consumption greater than 10%. Over age 80 there is a reduction of consumption in both men and women (Figure 2).

## Discussion

This study has some limitations that need to be clarified: all clinical data were automatically and anonymously obtained from electronic records, which are known to have large accuracy and completeness variability, as recently discussed by Majeed *et al *[7]; a systematic review showed the highest rate of recording for prescriptions, while morbidity was coded in 66% to 99% of consultations. Coronary heart disease was the most commonly assessed and completely recorded disease (around 70%), with a positive predictive value around 83–100%. In our case, registration of cardiovascular diseases and control variables has been continuously monitored and incentivized since 2006, with a set of clinical indicators to measure quality and outcomes. Nevertheless, measurement of data quality in electronic records is limited at the moment, because of a lack of reference standards for reporting data quality in primary care. This should be taken into account, as discussed later, when generalizing the results.

All patients with IHD were included, so recently diagnosed patients may not have had time to reach targets, for both men and women. This might represent a selection-bias, but would not affect the gender differences, when present.

As for results, they cannot be generalized to patients with IHD but not covered by a public health system which provides comprehensive health care and drug coverage as well as equality of access to sanitary services through a gate keeper.

The global prevalence of IHD diagnosis in this study population was 1.32%. This percentage increased with age, reaching 7% in subjects over 70. These figures are lower than those found in other studies in our geographical area (i.e. 5.5% in Barcelona, Baena *et al*) [8]. One cause for the differences may be that our study used patients' electronic medical records, registered by primary care clinicians during patient consultations, with a possible bias toward acute versus chronic presentations of disease, and towards more symptomatic patients among both men and women. Patients with angina only were not distinguished from patients with angina and AMI or AMI without angina, given that this was not the objective of the study. All patients affected by any clinical form of IHD were included, understanding that the secondary prevention measures studied are indicated for all IHD presentations. On the other hand, patients with IHD but without a registered diagnosis went undetected and were not included in the study. Obviously, these results can not be generalized to all patients with IHD, but only identified patients for whom management is the responsibility of primary care clinicians.

The fact that the study population was entirely drawn from recorded IHD diagnoses in one city means the group analyzed was homogenous. Geographic characteristics and the urban setting were the same. Available health care services were similar because there is a single set of primary care, hospital, and specialist treatment available to all residents, so differences can't be explained by these variables.

The study population had almost twice as many men as women, something seen in previous studies such as PRESENAP – conducted in Spain in 2004 with a sample of 8817 patients with IHD, of whom 74% were men [9]. PRESENAP also observed that men presented this disease at a younger age than women; our results show a mean age for women seven years greater than that of men, following the pattern described in the medical literature.

Chronic ischemic heart disease is the most frequent diagnosis registered in both sexes. The most frequent second diagnosis for men is acute myocardial infarct (23.3% vs. 17% in women), while the second most common diagnosis in women is *angor pectoris *(22.6% vs. 16% in men). The Framingham Heart Study describes the initial presentation of IHD in women as angina and in men as AMI. This, together with the greater proportion of normal coronariographies in women, results in IHD being considered more benign in women [10] and may have contributed creating a gender stereotype with a negative influence on management and prevention.

When analyzing screening, prevalence and management of modifiable CVRF, the following points stand out.

Screening for some CVRFs was high: BP and smoking behaviour were determined in more than 85% of patients, while BMI was calculated in 72.5% of cases, and cholesterol determined in 69.8%. On the other hand, only 62% of patients had glycemia tests registered during the year of the study. One recently published UK study by Crilly *et al *[5], done in a primary care setting with 1162 patients with angina, reported similar screening figures, greater than 85% for BP and smoking, and 72% for BMI. Screening percentages were similar between men and women, reflecting a growing sensitivity towards gender independent prevention. Men had slightly more frequent screening for hypertension, BMI and smoking (between 2.1% and 3.4% greater), and women slightly more screening for glycemia and cholesterol (between 3% and 3.8% greater). These differences, however, were not statistically significant.

The case of CVRF prevalence is different, with a greater percentage of women than men being hypertensive (70.8% vs. 57.1%, p < 0.01), obese (27.3% vs. 21.9%, p < 0.01) and diabetic (36.8% vs. 32.4%, p > 0.01). Comparing our results with other studies conducted in our area in patients of both sexes with IHD, such as the PRESENAP study mentioned earlier (HTN 76.6%, DM 32.7%), or with the Spanish data from EUROASPIRE II study (Europe, primary care, 2000: HTN 42.6%, obesity 34.1%, DM 39.2%) [11], our prevalence percentages are lower than expected. These differences have been attributed to diagnoses being obtained exclusively from coded registries in medical records generated by clinicians attending patients and not the results of complementary tests directly (BP, glycemia, BMI or cholesterol). The deficiencies in data entry in medical and hospital discharge records have been discussed in the earlier EUROASPIRE II study, in all participating European countries and at both primary and secondary care levels, suggesting that hospital discharge reports should include CVRF histories, CVRF measurements, prescribed treatments and management objectives for patients. However, as expected, these numbers confirm the high prevalence of CVRF in the IHD population with respect to the general population in our area, rates which are double that of the general population for HTN or obesity, and triple for DM, as reported by Marin *et al *in 2006 after five years of primary care follow-up of a cohort of 6124 patients in Zaragoza [12].

Smoking, on the other hand, was much more common in men than women (19% vs. 3.1%), coinciding with previously published figures [9]. It was the only CVRF which was less frequent in the IHD population than in the general population, reflecting, on one hand, the effect of advanced age and, on the other, the tendency to stop smoking when diagnosed with IHD. Nonetheless, one out of five men continued to smoke.

The last factor studied, dyslipemia, affected both sexes equally, at about 44%. Disparities with diagnostic criteria used in other studies prevented comparison with our figures.

When analyzing the management of these CVRF in function of current recommendations, only 52.1% of IHD patients had their BP below 140/90, with a slight predominance of men (3.7% more men properly managed, p > 0.01). Other studies have reported highly variable rates for this risk factor: 74.6% of the control group in the PRESENAP study (Spain, primary care, 2004), 49.6% in the l'EUROASPIRE II study (Europe, primary care, 2000) or 38% in the NHANES III study (USA, primary care, 1994) [13].

The percentage of patients that maintained a LDL level below 100 mg/dl was roughly 38.6%: the PRESENAP study reported 26.3% and the NHANES III study reported 49%. The percentage of men with well-managed LDL was greater than that of women by 3.6%, with no statistically significant difference.

For diabetes, glycosylated hemoglobin was below seven in 34.4% of our cases, with 2% more men well managed with respect to women (p > 0.01). This figure was 50.6% in PRESENAP and 47% in NHANES III.

Poorer management was seen with regard to weight. Only 10.6% of all patients maintained a BMI below 25, with worse results in women, even though the difference was minimal (1.4% p > 0.01).

In sum, although control of these CVRF was better in men, the observed differences related to risk management were not statistically significant.

Overall, CVRF management was poor in this group of patients with IHD; the percentages would be even lower when considering some of these CVRF simultaneously. These poor results are not explained by lack of information, because screening for CVRF was high.

The current recommendations for secondary prevention of cardiovascular disease focus on the prescription of anticoagulants, beta blockers, ACE inhibitors/ARB and lipid-lowering agents. The percentage of prescriptions in the study population were greater than those reported in other studies for beta-blockers and lipid-lowering agents, and less for anticoagulants: the cited work of Crilly *et al *refers to 35% for prescriptions of beta-blockers and 55% for statins compared to 48.6% and 64%, respectively, in our study population. Antiplatelet/anticoagulant drugs were prescribed in 84% of their cases while in our study the figure was 80% [5].

Men in our study population had a greater probability of receiving medications from any of the four pharmaceutical groups than women, and this relationship tended to persist when adjusting for age. Studying the four types of medications individually by age groups and by IHD specific diagnosis, there continued to be differences, which were significant for anticoagulant/antiplatelet drugs and lipid-lowering agents. There is no evidence to suggest that secondary prevention is more effective in men than in women, so there should not be any gender-related differences in the results. Our findings are consistent with those described in other studies that analyzed gender-related inequalities in health ([2,4] and [14]) and suggest that clinical diagnosis (*angor pectoris*, AMI, chronic IHD) may be related to these differences, taking into account that women tend to present more frequently with *angor pectoris *than men, and this may condition the quantity and quality of treatment. It is possible that men receive more anticoagulant medication because they present more frequently with AMI than with angina, and more lipid-lowering agents because of the perception that IHD is more serious in them. On the other hand, these differences disappear for ACE inhibitors/ARB and beta-blockers when adjusted for diagnosis, probably because hypertension is more common in women and these drugs are widely used for its treatment.

Other gender-related variables were not available and not examined, such as co morbidity, which may influence the decision of whether to prescribe a given medication to a given patient, acting as confounding factors. All these hypotheses need to be examined in studies specifically designed to address them.

## Conclusion

There were no important observed differences between men and women with IHD related to CVRF screening. On the whole, recorded screening activity can be considered elevated for HTN and smoking, and needing improvement with regard to obesity, dyslipemia and diabetes.

The prevalence of these factors is consistent with the expected values for this population, with HTN, DM and obesity being the most frequent among women.

Adequate management of CVRF tended to be slightly worse in women, with no statistically significant differences. Overall, it is important to note the poor control of CVRF in patients with established heart disease, both men and women. Primary care physicians as well as hospital specialists should be aware of these findings and consider the reasons for this deficiency.

Gender-related inequalities were seen in medications provided for secondary IHD prevention. Men had a greater probability of receiving any of the recommended medications and this result persisted across all age groups, a difference which is not justified by the currently available evidence, since the demonstrated beneficial effects of these treatments on the evolution of disease are the same regardless of the sex of the patient.

As a final conclusion, our findings confirm that gender differences are still found in the management of patients with IHD: attention has to focus on women in order to detect avoidable gender-bias, and consequently, worse health results.

## Competing interests

The authors declare that they have no competing interests.

## Authors' contributions

MCS conceived the study, participated in its design and helped to draft the manuscript. IC participated in the design and drafted the manuscript. JR performed the statistical analysis, interpreted the results and helped to draft the manuscript. GG and EG participated in the design, interpreted the results and helped to draft the manuscript. LG provided, validated and helped to interpret pharmacological data.

All authors read and approved the final manuscript.

## Funding

None.

## Pre-publication history

The pre-publication history for this paper can be accessed here:


